# Hearing Impairment With Cognitive Decline Increases All-Cause Mortality Risk in Chinese Adults Aged 65 Years or Older: A Population-Based Longitudinal Study

**DOI:** 10.3389/fnagi.2022.865821

**Published:** 2022-06-24

**Authors:** Jun Wang, Dan Liu, E. Tian, Zhao-Qi Guo, Jing-Yu Chen, Wei-Jia Kong, Su-Lin Zhang

**Affiliations:** ^1^Department of Otorhinolaryngology, Union Hospital, Tongji Medical College, Huazhong University of Science and Technology, Wuhan, China; ^2^Institute of Otorhinolaryngology, Union Hospital, Tongji Medical College, Huazhong University of Science and Technology, Wuhan, China

**Keywords:** hearing impairment, cognitive impairment, mortality, cohort study, aging

## Abstract

**Background:**

Hearing impairment (HI), a highly prevalent sensory impairment affecting older adults, is a risk factor for cognitive decline. However, few studies examined the association between HI and all-cause mortality, and the role of different cognitive states on this relationship in Chinese older adults is poorly understood.

**Methods:**

A total of 10,744 Chinese older adults aged 65 years or older were included in the 2011/2012 and 2014 cohorts from the Chinese Longitudinal Healthy Longevity Survey (CLHLS), with the longest follow-up period lasting for up to 8 years. The presence of HI was identified by using a dichotomized metric of self-reported hearing status. All-cause mortality data were ascertained from interviews with family members or relatives of the participants. Cognitive function was evaluated by employing the modified Mini-Mental State Examination (MMSE), which consisted of seven subdomains (orientation, naming foods, registration, attention and calculation, copy figure, delayed recall, and speech and language). Kaplan–Meier survival curves were constructed to evaluate the different hearing states on overall survival. The risk of mortality over the follow-up period was estimated by using Cox proportional hazard ratios (HRs) models.

**Results:**

A conspicuous probability was revealed in the survival relationship between hearing status and all-cause mortality for the total population (*p* < 0.001). Participants with HI had a higher risk of all-cause mortality (HR = 2.29, 95% CI: 2.16, 2.42), as compared with their counterparts without HI. The association was robust upon fully adjustment for potential confounders (HR = 1.07, 95% CI: 1.00, 1.14). Compared to HI participants with no cognitive impairment, HI patients with cognitive impairment had a higher mortality risk (HR = 2.31, 95% CI: 2.13, 2.51). Impairment in the subdomains of cognitive function were independently associated with elevated mortality risk in the participants with HI, with an HR ranging from 1.28 (copy figure) to 1.46 (speech and language).

**Conclusions:**

Cognitive decline was common in individuals with HI, and those with HI and cognitive impairment further increased mortality risk. Our findings prompt a call for actions to improve the hearing status and cognitive function of older people to minimize health risks and improve longevity.

## Introduction

Population aging represents the single most substantial demographic change of the 21st century, stemming from the decline in both fertility and mortality rates. The global number of people aged 60 years or older is projected to increase from 970 million to 2.1 billion in 2050 and 3.1 billion in 2100 (Ganesan et al., 2019). About 80% of the aging population will be in the developing countries (Ganesan et al., 2019). Population aging has resulted in a notable epidemiological transition, characterized by an increased prevalence of chronic diseases, including hearing impairment (HI) and cognitive impairment (Davis et al., 2016; Vancampfort et al., 2017; Ganesan et al., 2019).

The HI has become a common handicap across the globe (Brown et al., 2018) and as an age-related disease, HI imposes the burden on the society at large. A recent report by the *Annals of Internal Medicine* estimated that while two thirds of Americans aged 70 years or over have HI, only 15–20% of United States older adults use hearing aids, and disparities exist by ethnicity and socioeconomic status (Nieman and Oh, 2020). Similarly, in 2014–2015 a field survey in four representative provinces in China found that the prevalence rate of HI was approximately two thirds among Chinese adults aged 60 years or older (Gong et al., 2018). Etiologies of HI are multifactorial (Wang and Puel, 2020), involving genetic, chronic infectious, noise-induced, ototoxic (particularly iatrogenic ototoxicity), traumatic, immune-mediated, and age-related causes, among others. In addition, many people regard HI as a natural process of aging that can be ignorable. As a result, HI has not yet received enough attention it deserves. Previous studies reported that HI might be a modifiable condition and a possible target for secondary prevention of cognitive impairment in older age, dementia, social isolation, late-life depression, frailty and increased risk of mortality (Deal et al., 2017; Brenowitz et al., 2019; Wang and Puel, 2020). Further research is warranted to determine whether extensive hearing rehabilitative interventions could delay or halt cognitive decline and thereby lower the risk of mortality.

Cognitive impairment is among the most pressing public health concerns worldwide and has recently been found to be associated with HI (Lin and Albert, 2014; Martini et al., 2014; Loughrey et al., 2018; Powell et al., 2021). Previous studies exhibited that HI accelerated cognitive decline (Powell et al., 2021). However, the hearing decline is gradual and tends to go unrecognized, consequently, receives minimal attention (Michikawa et al., 2009). Gao et al. (2020), in their study of the CLHLS data sets of 2011/2012 and 2014 waves, confirmed that HI was negatively associated with cognitive function in older adults in China. The mechanism underlying the association between HI and cognitive impairment remains unclear, several postulations were proposed (e.g., information-degradation, sensory deprivation, and common pathologic etiology) (Lin and Albert, 2014; Martini et al., 2014; Loughrey et al., 2018; Powell et al., 2021). In addition, previous studies did not examine how HI, with or without cognitive impairment, impacts the all-cause mortality.

The relationship between HI and cognitive function and mortality is complex. Although the relationship between HI and mortality risk has been studied in the populations in high-income countries and the results were inconsistent (Genther et al., 2015; Engdahl et al., 2019; Lin et al., 2019; Miyawaki et al., 2020; Sun et al., 2020). However, the evidence from low- and middle-income countries are far from sufficient, because these countries are experiencing the fastest rise in life expectancy. In addition, a number of epidemiological studies have demonstrated an association between cognitive impairment and increased risk of mortality, with both mild and moderate-to-severe cognitive impairment being predictors of mortality in older people (Takata et al., 2014; An and Liu, 2016; Lv et al., 2019; Li et al., 2021). Nevertheless, previous studies on the association between HI and mortality did not examine the potential differences in the cognitive function and its subdomains in the older people, and further research is needed (Rabbitt, 1990; Baltes and Lindenberger, 1997; Dawes et al., 2015). Therefore, if HI and cognitive decline can serve as predictors of mortality, they should be studied to understand their impact on the mortality burdens.

In the present study, we used longitudinal cohort study data from the most recent 2011/2012 and 2014 CLHLS waves of follow-up to estimate (i) the association between time-varying HI and all-cause mortality among the oldest-old; (ii) the potential role of cognitive function and its subdomains in this relationship.

## Materials and Methods

### Data Sources and Study Cohort

The present study took data from the Chinese Longitudinal Healthy Longevity Survey (CLHLS), which is an ongoing longitudinal study that began in 1998, along with seven follow-up surveys, with the new participants being added to replace the deceased who passed away in 2000, 2002, 2005, 2008, 2011/2012, 2014, and 2018 (Yang and Meng, 2020). The CLHLS recruited a representative sample from 23 of the 31 provinces of China and is the largest database on the oldest-old in the world, with the survey areas covering 85% of the Chinese population (Zeng, 2012). More details about the sampling procedure and quality of data of this survey have been published elsewhere (Zeng, 2012). Ethics approval was obtained from the Research Ethics Committees of Peking University (IRB00001052-13074). All participants or their legal representatives signed written consent forms in the baseline and follow-up surveys.

The CLHLS consisted of questions regarding self-reported hearing difficulties only in waves 2011/2012 and 2014. Thus, this study employed two waves from the CLHLS longitudinal data harvested during 2011/2012 to 2014. A total of 10,890 participants were enrolled, including 10,744 aged 65 years or older, with the latest follow-up conducted in 2018. Accordingly, 9,674 respondents were interviewed in 2011/2012 wave, and 1,070 newly enrolled respondents were interviewed in 2014 wave, respectively. Data were incomplete for 1,703 participants, and the amount of missing data (key variables) ranged from 54 to 1,005. The longest follow-up period lasted for up to 8 years, and 47.5% (*n* = 5,099) of the participants died during the follow-up period until 2018. Details of the sampling method and calculation of weights have been published previously (Dawes et al., 2015). Characteristics of the raw dataset were shown in Supplementary Figure 1.

### Assessment of Hearing Status

Hearing sensations were assessed in terms of self-reported measures, and all enrolled participants were required to attend a series of standardized training sessions prior to interviews. Self-reported hearing status data were based on the responses to the question: “Do you have any difficulty with your hearing? “YES” was coded as having HI, while “No” signified not having HI (Gao et al., 2020).” A systematic review compared the results obtained with self-report to a hearing question with those obtained by pure tone audiometry. They found that older adults with HI can be recommended for an epidemiologic study if audiometric measurements cannot be performed (Valete-Rosalino and Rozenfeld, 2005). Therefore, self-reported hearing status is a suitable option for large epidemiological studies (Valete-Rosalino and Rozenfeld, 2005; Deepthi and Kasthuri, 2012; Diao et al., 2014).

### Assessment of Cognitive Function

**FIGURE 1:**
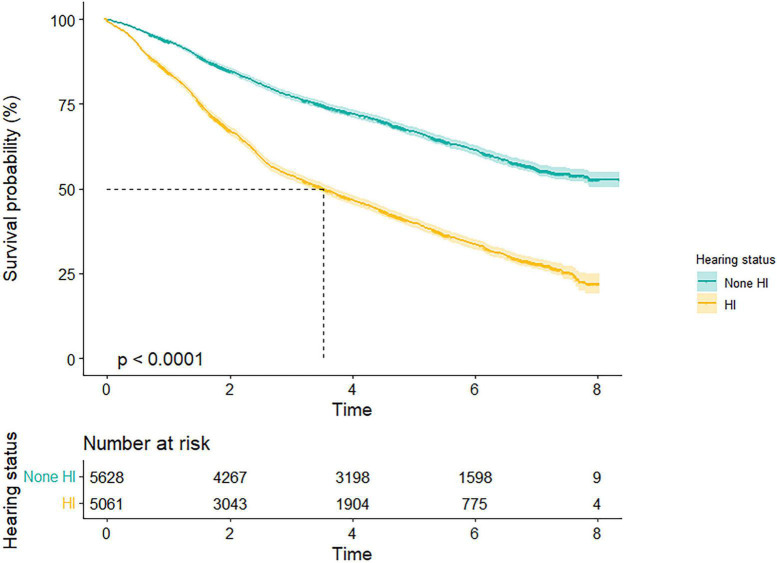
Survival probability by different hearing states. Kaplan–Meier survival curves illustrated a conspicuous probability of survival relationship between hearing status and all-cause mortality for total population (*p* < 0.001). HI, hearing impairment.

All participants were assessed for cognitive function by utilizing the Chinese version of the Mini-Mental State Exam (MMSE), a widely used cognitive test (Christensen et al., 2013). The test was tailored to the Chinese language based on the international standard of MMSE questionnaire, and had been proven to be reliable and valid in previous studies (Yi and Vaupel, 2010; Yuan et al., 2019; Duan et al., 2020; Zhang et al., 2021). The Chinese version of MMSE evaluates cognitive function in terms of 24 items, covering 7 sub-scales: orientation (4 points for time orientation and 1 point for place orientation); naming foods (naming as many kinds of food as possible in 1 min, 7 points); registration of 3 words (3 points); attention and calculation (mentally subtracting 3 iteratively from 20, 5 points); copy a figure (1 point); recall (delayed recall of the 3 words mentioned above, 3 points); and speech and language (2 points for naming objectives, 1 point for repeating a sentence, and 3 points for listening and following directions). The MMSE score ranges from 0 to 30. The higher the score, the better the cognitive function. The individuals who scored 25 or higher were considered to have normal cognitive function, and the summary accuracy at a cutoff value of 25 (10 studies) was sensitivity 0.87 and specificity 0.82 (Creavin et al., 2016). Cognitive impairment in this study was determined by its presence or absence according to this classification (Creavin et al., 2016).

### Data on Mortality

Information on mortality was collected on the basis of death certificates provided by the local authorities. When such information was not available, relatives of the decedents were interviewed. Duration of follow-up was the time interval from the first interview date until the date of death. Participants who were alive at the last interview were regarded as being censored on the dates of their last interviews in 2018. The cause-specific mortality was not involved in this study because (1) a lot of the older adults died at home rather than in medical institutions where cause of mortality might be recorded, and (2) mortality surveillance systems are unsure in many survey fields (Duan et al., 2020).

### Assessment of Potential Confounding Variables

A variety of variables were collected through a face-to-face interview against a standardized questionnaire, including sociodemographic features, lifestyles, health conditions and available medical services that were potentially associated with HI and cognitive impairment as suggested by previous studies (Dawes et al., 2015; Loughrey et al., 2018; Lv et al., 2019). Therefore, in this study, we assessed a range of these potential confounders by including the covariates age (continuous), gender (male or female), education background (no schooling or primary school or higher), occupation before retirement (manual or non-manual), ethnicity (Han or minority), residence (urban or rural), marital status (currently married and living with spouse, separated/divorced/never married, or widowed), tobacco smoking status (never or ever), alcohol drinking status (never or ever), regular leisure activities (yes or no), activity of daily living (ADL) (don’t need help or need help), having been diagnosed with hypertension (yes or no), having been diagnosed with diabetes (yes or no), and baseline cognitive function (impaired or not impaired).

### Statistical Analysis

First, we used multiple imputation to impute missing data for our raw dataset. The multiple imputation is based on chained equations and is commonly used for longitudinal studies. As multiple imputation uses information on baseline demographics and previous time points to predict missing values, the strategy assumes that data are missing at random, that is, that the missingness is related to observed data. We used some demographics such as gender, age, place of residence, as predictors, to impute the missing values of key variables. We established 10 imputed datasets and carried out the pooled statistical inference.

For comparison, Chi-square test was used for categorical variables and the analysis of variance was employed for continuous variables. The Kaplan–Meier method was employed to plot the survival curves in terms of baseline hearing status and gender. Multivariable-adjusted hazard ratios (HRs) and 95% confidence intervals (CIs) of all-cause mortality by hearing status were calculated by using five dependent Cox proportional hazards models: Model 1: no variables adjusted; Model 2: additionally adjusted for gender, age, education background, residence, and marital status based on model 1; Model 3: additionally adjusted for smoking status, drinking status, regular leisure activities, and ADL based on model 2; Model 4: additionally adjusted for two kinds of diseases (hypertension and diabetes) based on model 3; and Model 5: additionally adjusted for baseline cognitive function based on model 4. Our test ascertained that the proportional hazard assumption was not been violated.

Next, in order to assess disparities across different populations, we conducted subgroup analyses in terms of baseline cognitive function (MMSE score ≥25 versus <25 points), age (65–79 versus ≥80 years), and gender (female versus male), respectively. In addition, we also examined whether the association of HI with mortality differed by baseline cognitive function and gender by separately adding an interaction term to the fully adjusted model. Moreover, we further examined the association of HI with all-cause mortality by seven cognitive subdomains among Chinese older adults.

In the end, to resolve the problem with the loss to follow-up, we performed a sensitivity analysis by removing incomplete cases. Additionally, we also conducted another sensitivity analysis by eliminating those who died within the half of the year after the baseline survey to account for the possibility that the pre-mortality dropped in hearing function and/or disease status could have influenced our results.

A two-tailed *p*-value of less than 0.05 was considered statistically significant. All analyses were performed by using R software package (version 4.1.1, the R Foundation for Statistical Computing, Vienna, Austria).

## Results

### Participant Characteristics

Table 1 presents the descriptive baseline characteristics in terms of different hearing states. A total of 10,744 enrolled participants aged 65 or older participated in the baseline survey in waves during 2011/2012 and 2014, and they were followed up for at least one wave. All participants were aged 86 years on average (range: 65–114 years) and more than half of them (55.5%) were female. Participants with HI more likely to be older, female, lower-educated, rural residents, widowed, separated, divorced or never married, no regular leisure activities, need help in ADL, having had hypertension and diabetes, and worse cognitive function. However, participants with HI were less likely to smoke and drink, but were more likely to have disease conditions (Table 1).

**TABLE 1:** Baseline characteristics by hearing status among Chinese aged ≥65 years.

	Hearing status^a^			
	None-HI	HI	Total	*P-*value^e^
***N* (%)**	5665 (52.7)	5079 (47.3)	10744	
**Age, years, median (25th, 75th)**	81 (73, 89)	92 (84, 99)	86 (77, 95)	<0.001
**Gender, count (%)**				<0.001
Male	2724 (48.1)	2053 (40.4)	4777 (44.5)	
Female	2941 (51.9)	3026 (59.6)	5967 (55.5)	
**Education attainment^b^, count (%)**				<0.001
No schooling	2881 (50.9)	3472 (68.4)	6353 (59.1)	
Primary school or higher	2784 (49.1)	1607 (31.6)	4391 (40.9)	
**Main occupation before age 60, count (%)**				<0.001
Non-manual	1016 (17.9)	617 (12.1)	1633 (15.2)	
Manual	4649 (82.1)	4462 (87.9)	9111 (84.8)	
**Ethnicity, count (%)**				0.261
Han	5237 (92.4)	4791 (94.3)	10028 (93.3)	
Others (minority)	428 (7.6)	288 (5.7)	716 (6.7)	
**Residence, count (%)**				0.001
Urban	2611 (46.1)	2285 (45.0)	4896 (45.6)	
Rural	3054 (53.9)	2794 (55.0)	5848 (54.4)	
**Marital status, count (%)**				<0.001
Currently married and living with spouse	2616 (46.2)	1216 (23.9)	3832 (35.7)	
Others^c^	3049 (53.8)	3863 (76.1)	6912 (64.3)	
**Tobacco smoking status, count (%)**				<0.001
Never	3689 (65.1)	3564 (70.2)	7253 (67.5)	
Ever	1976 (34.9)	1515 (29.8)	3491 (32.5)	
**Alcohol drinking status, count (%)**				0.001
Never	3903 (68.9)	3647 (71.8)	7550 (70.3)	
Ever	1762 (31.1)	1432 (28.2)	3194 (29.7)	
**Regular leisure activities, count (%)**				<0.001
Yes	3444 (60.8)	1679 (33.1)	5123 (47.7)	
No	2221 (39.2)	3400 (66.9)	5621 (52.3)	
**ADL, count (%)**				<0.001
Don’t need help	4752 (83.9)	3115 (61.3)	7867 (73.2)	
Need help	913 (16.1)	1964 (38.7)	2877 (26.8)	
**Self-reported hypertension, count (%)**				<0.001
With	1803 (31.8)	1399 (27.5)	3202 (29.8)	
Without	3862 (68.2)	3680 (72.5)	7542 (70.2)	
**Self-reported diabetes, count (%)**				0.001
With	288 (5.1)	192 (3.8)	480 (4.5)	
Without	5377 (94.9)	4887 (96.2)	10264 (95.5)	
**Cognitive function^d^, count (%)**				<0.001
Impaired	1412 (24.9)	3081 (60.7)	4493 (41.8)	
Not impaired	4253 (75.1)	1998 (39.3)	6251 (58.2)	

*HI, hearing impairment; ADL, activities of daily living.*

*^a^Hearing status was defined by responses of self-reported hearing status: none-HI and HI.*

*^b^Education background was defined by education attainment. None: school years = 0; primary school: school years = 1–5; middle school or higher: school years > 5.*

*^c^‘Others’ include widowed, separated, divorced and never married.*

*^d^Cognitive function was classified into two mutually exclusive groups: not impaired (25 ≤ MMSE score ≤ 30), and impaired (0 ≤ MMSE score ≤ 24).*

*^e^Chi-square test was used for categorical variables, and analysis of variance was used for continuous variables.*

### Kaplan–Meier Curves and Results of Multivariable Analysis in All Participants

Kaplan–Meier survival curve revealed a conspicuous probability of survival relationship between hearing status and all-cause mortality for total population (log-rank test for trend: *p* < 0.001) (Figure 1). The median survival time of none-HI and HI participants were 4.7 and 2.7 years, respectively, among the total population.

Table 2 shows the multivariable-adjusted HR and 95% CI of all-cause mortality by hearing status among Chinese older adults aged ≥65 years. The five multivariate models displayed consistent patterns between HI and all-cause mortality, and the association attenuated with more covariates included in the models. The unadjusted model (Model 1) showed that HI was significantly associated with the all-cause mortality. Compared to participants without HI, those who reported HI during the follow-up period were 2.29 times more likely to have all-cause mortality (95% CI: 2.16, 2.42). After adjustment for gender, age, education background, residence, and marital status in Model 2, the association between HI and mortality diminished significantly but remained comparable, with HR = 1.28 (95% CI: 1.20, 1.36). Model 3 (HR = 1.14, 95% CI: 1.07, 1.21) showed that HR decreased from 1.28 to 1.14 (i.e., 14% hazard ratio reduction) after adjustment for smoking, drinking, regular leisure activities, and ADL based on Model 2. Model 4 was additionally adjusted for two kinds of diseases (hypertension and diabetes) based on Model 3, and the results were consistent with Model 3. Model 5 was additionally adjusted for baseline cognitive function based on model 4, and the results suggested that HI was associated with a 7% [HR = 1.07, (95% CI: 1.00, 1.14)] increase in the risk of all-cause mortality compared with individuals without HI.

**TABLE 2:** Multivariable-adjusted hazard ratios and 95% confidence intervals of all-cause mortality by hearing status.

Model	Model 1	Model 2	Model 3	Model 4	Model 5
	
	Hazard ratio (95% CI)				
**Hearing impairment**					
No	–	–	–	–	–
Yes	2.29 (2.16, 2.42)	1.28 (1.20, 1.36)	1.14 (1.07, 1.21)	1.14 (1.07, 1.21)	1.07 (1.00, 1.14)
**Gender**					
Female	–	–	–	–	–
Male		1.42 (1.33, 1.52)	1.43 (1.33, 1.54)	1.43 (1.33, 1.54)	1.46 (1.35, 1.57)
**Age**	–	1.07 (1.07, 1.08)	1.05 (1.05, 1.06)	1.05 (1.05, 1.06)	1.05 (1.05, 1.06)
**Education attainment**					
Primary school or higher	–	–	–	–	–
None		1.14 (1.06, 1.22)	1.08 (1.01, 1.16)	1.08 (1.01, 1.16)	1.04 (0.97, 1.12)
**Residence**					
Rural	–	–	–	–	–
Urban		1.04 (0.99, 1.10)	1.07 (1.01, 1.13)	1.07 (1.01, 1.13)	1.05 (0.99, 1.11)
**Marital status**					
Currently married and living with spouse	–	–	–	–	–
Others^a^		1.25 (1.16, 1.35)	1.19 (1.10, 1.28)	1.19 (1.10, 1.29)	1.17 (1.08, 1.26)
**Smoke status**					
Never	–	–	–	–	–
Ever			1.11 (1.03,1.19)	1.10 (1.03,1.19)	1.13 (1.05, 1.21)
**Drink status**					
Never	–	–	–	–	–
Ever			1.01 (0.95, 1.08)	1.01 (0.95, 1.08)	1.01 (0.94, 1.08)
**Regular leisure activities**					
Yes	–	–	–	–	–
No			1.78 (1.66, 1.90)	1.77 (1.65, 1.90)	1.65 (1.54, 1.77)
**ADL**					
Don’t need help	–	–	–	–	–
Need help			1.71 (1.61, 1.82)	1.71 (1.60, 1.82)	1.60 (1.50, 1.70)
**Self-reported hypertension**					
Without	–	–	–	–	–
With				0.98 (0.92, 1.04)	0.98 (0.92, 1.04)
**Self-reported diabetes**					
Without	–	–	–	–	–
With				1.34 (1.17, 1.54)	1.33 (1.16, 1.53)
**Cognitive function**					
Not impaired	–	–	–	–	–
Impaired					1.47 (1.38, 1.58)

*CI, confidence interval; ADL, activities of daily living.*

*Model 1: No variables adjusted.*

*Model 2: Additionally adjusted for gender, age, education attainment, residence, and marital status based on model 1.*

*Model 3: Additionally adjusted for smoking status, drinking status, regular leisure activities, and ADL based on model 2.*

*Model 4: Additionally adjusted for two kinds of diseases (hypertension and diabetes) based on model 3.*

*Model 5: Additionally adjusted for cognitive function based on model 4.*

*^a^‘Others’ include widowed, separated, divorced and never married.*

### Subgroup Analyses

**FIGURE 2:**
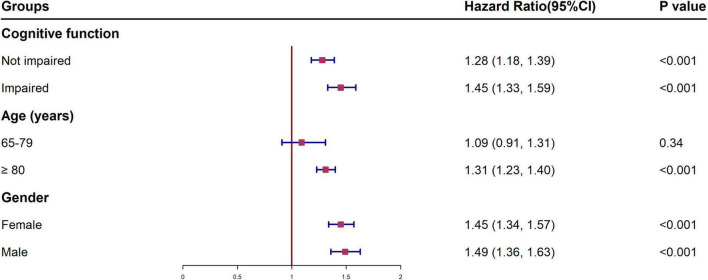
Multivariable-adjusted hazard ratios and 95% confidence intervals of all-cause mortality for different subgroups. ^†^All models were adjusted for gender, age education, residence, and marital status, smoking status, drinking status, regular leisure activities, ADL, hypertension and diabetes. HI, hearing impairment; HR, hazard ratio; CI, confidence interval; ADL, activities of daily living.

**FIGURE 3:**
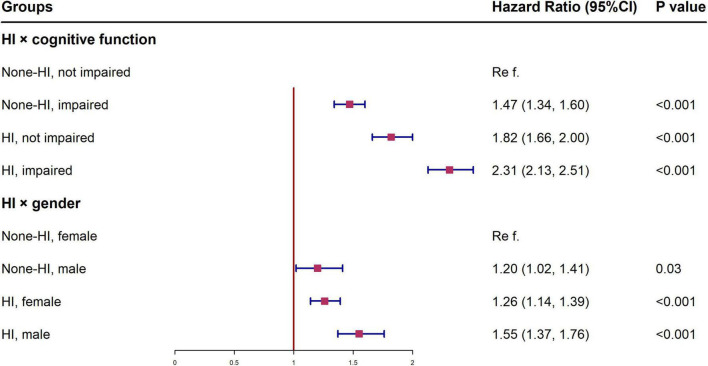
Interactions between hearing status and cognitive function, gender in the association with all-cause mortality among Chinese aged ≥65 years. ^†^All models were adjusted for gender, age, education, residence, and marital status, smoking status, drinking status, regular leisure activities, ADL, hypertension and diabetes. HI, hearing impairment; CI, confidence interval; ADL, activities of daily living.

Figure 2 shows the HR of all-cause mortality by HI for different subgroups, conducted as separate models for each subgroup with full adjustment as in Model 5. The relationship and the effect sizes between HI and all-cause mortality were consistent across all subgroups in terms of cognitive function, age and gender. To examine whether the association between HI and all-cause mortality is modified by different cognitive states, we further tested the interaction between hearing status and cognitive function. The results showed that there was a significant interaction between HI and cognitive impairment (*p* < 0.001). HI participants with cognitive impairment showed higher mortality risk (HR = 2.31, 95% CI: 2.13, 2.51) than other groups (Figure 3). Similar associations were observed in the interaction analysis based on hearing status and gender. We also found that males had a higher risk of mortality (HR = 1.55, 95% CI: 1.37, 1.76) than their female counterparts, when they had the same level of HI (Figure 3).

### Subdomain Analysis

We examined the seven cognitive subdomains separately for the association between HI and all-cause mortality. Impairment in orientation, naming foods, registration, attention and calculation, copy figure, delayed recall, and speech and language was independently associated with elevated mortality risk in the participants with HD, with HR ranging from 1.28 (copy figure) to 1.46 (speech and language) (Table 3).

**TABLE 3:** Association of hearing impairment with all-cause mortality by different cognitive function subdomains^a^.

Subdomains^b^	Hazard ratio (95% CI)	*P-*value
**Orientation**		<0.001
Not impaired	–	
Impaired	1.43 (1.34, 1.52)	
**Naming foods**		<0.001
Not impaired	–	
Impaired	1.31 (1.23, 1.39)	
**Registration**		<0.001
Not impaired	–	
Impaired	1.37 (1.29, 1.46)	
**Attention and calculation**		<0.001
Not impaired	–	
Impaired	1.32 (1.24, 1.40)	
**Copy figure**		<0.001
Not impaired	–	
Impaired	1.28 (1.18, 1.39)	
**Delayed recall**		<0.001
Not impaired	–	
Impaired	1.35 (1.27, 1.43)	
**Speech and language**		<0.001
Not impaired	–	
Impaired	1.46 (1.36, 1.55)	

*HI, hearing impairment; CI, confidence interval; MMSE, mini-mental state examination; ADL, activities of daily living.*

*^a^Measured by the MMSE, and each of the seven cognitive subdomains was dichotomized with full score indicating no impairment and all others as impaired.*

*^b^Each model was adjusted for all the covariates, including demographics (gender, age, education, residence, and marital status), and smoking status, drinking status, regular leisure activities, ADL, hypertension and diabetes.*

### Sensitivity Analysis

Among the cohorts, there was almost no change in the association between HI and all-cause mortality after excluding participants lost to follow-up or with survival time less than half of the year. The association was still robust after further adjustment for potential confounders (Supplementary Tables 1, 2).

## Discussion

With the population aging, an increasing number of people are living with HI, especially during their later-life years, which can bring about multiple health problems. To our knowledge, this is the first longitudinal survey to examine if HI bears a relation with all-cause mortality and what role the cognitive function plays in Chinese adults aged 65 years or older. Subgroup and sensitivity analyses revealed that the associations remained robust.

Findings of the present study suggested that the prevalence of HI was 47.3% (59.6% women), with gender being highly correlated with HI. The incidence of HI in our cohort was similar to that in the United States and Western European countries (Roth et al., 2011; Tyagi et al., 2021). The Framingham Cohort Study (*n* = 1,672, mean age = 59 years, 57.6% women) (Tyagi et al., 2021) found that the prevalence of abnormal hearing patterns stood at 57.3% (i.e., 20.3% cochlear-conductive; 20.3% sensorineural; 7.7% low-sloping; and 8.0% strial). A review concluded that approximately 30% of men and 20% of women in Europe have a hearing loss of 30 dB or more by the age 70 years, and 55% of men and 45% of women by the age 80 years (Roth et al., 2011). Additionally, in line with previous studies (Feeny et al., 2012; Denney and Boardman, 2021), we found that the aging male participants with HI carried a higher risk of mortality than their female counterparts after adjusting for confounding factors. The gender differences might be ascribed to the following reasons: (i) males had a higher innate and pro-inflammatory activity and lower adaptive immunity (i.e., testosterone has an immunosuppressive effect while estrogen has an immunoenhancing effect on the immune system) (Taneja, 2018); (ii) males had a higher incidence of smoking and drinking in the CLHLS dataset, and these risk factors act as drivers for mortality risk, leading to the differences (Feeny et al., 2012). The finding reminds us that when the government and organizations are building programs to prevent hearing function, gender differences should not be ignored.

Consistent with previous studies (Takata et al., 2014; Lv et al., 2019; Li et al., 2021), our study, using the CLHLS database, yielded an important finding that self-perceived HI is associated with the risk for all-cause mortality independent of demographics, health behaviors, certain comorbidities, and baseline cognitive function. In a nationally representative dataset in United States, involving 215.6 million Americans (mean age = 45.9 years, 51.7% female), Lin et al. (2019) revealed a 5-year mortality rate of 3.0% in those with good hearing and a rate of 19.5% in participants with HI and a rate of 17.8% in deaf individuals. Genther et al. (2015) analyzed audiologic data from 1,958 adults aged 70–79 years from the Health, Aging, and Body Composition Study. They found a HR of 1.64 for mortality in individuals with HI, as compared to normal hearing individuals, and the association remained consistent (HR = 1.20, 95% CI: 1.03–1.41) when the effects were adjusted for demographics and cardiovascular risk factors. Our results demonstrating attenuation of the association of HI and mortality after adjustment for demographics and cardiovascular factors are consistent with these previous findings.

Our study revealed that poor cognitive performance was common in individuals with HI, and its interaction with cognitive impairment further increased mortality risk in older adults. Additionally, we also found that impairment in the subdomains of cognitive function was independently associated with increased mortality risk among participants with HI, especially in the subdomains of speech and language. Multiple assumptions have been put forward to explain the association between HI and cognitive impairment (Lin and Albert, 2014; Yamada et al., 2016; Powell et al., 2021). The first is the information-degradation hypothesis, which postulates that the increased cognitive load associated with HI adjustment may deplete available resources for performing other cognitive activities. The second is the sensory deprivation hypothesis: that is, HI leads to cortical re-allocation, deafferentation, or atrophy to support speech perception processing. The third hypothesis is a shared pathologic etiology: i.e., a common cause such as aging or microvascular disease may result in both HI and cognitive impairment. Based on the hypothesis model of HI and cognitive function proposed by Lin and Albert (2014), we further put forward a possible mechanism by which HI and cognitive impairment work on all-cause mortality (Supplementary Figure 2). Specifically, HI in the presence of cognitive impairment may serve as a marker for frailty (e.g., physical, cognitive, social, and psychological frailty) (Fried et al., 2001; Panza et al., 2018), which is a powerful predictor of mortality.

Our study has its own strengths and limitations. On the one hand, the power of this study lies in that it was a large nationally-representative cohort of the oldest-old population, with negligible loss to follow-up in terms of mortality. In addition, the protracted follow-up period enabled us to conduct in-depth subgroup and subdomain analyses upon adjustment for potential confounding variables. On the other hand, hearing sensations were assessed in terms of self-reported measures, which did not reflect the severity of HI. Audiometry is the gold standard for evaluation of hearing loss, but large-scale use of the procedure involves operational difficulties (Deepthi and Kasthuri, 2012; Diao et al., 2014). Additionally, our cognitive function depended on the MMSE. Albeit it has been validated in population-based studies, it is not a professional diagnosis of cognitive impairment (Christensen et al., 2013; Creavin et al., 2016). Finally, we did not have access to detailed information about the specific cause and duration of HI. Further studies are warranted to examine the relationship between HI and mortality in varied degrees and durations.

## Conclusion

The data from this population-based longitudinal study revealed a conspicuous probability of survival relationship between hearing status and all-cause mortality in Chinese aged 65 or older. The association remained robust in subgroup and sensitivity analyses. In addition, cognitive decline was common in individuals with HI, and its interaction with cognitive impairment further increased mortality risk in older adults. Our findings prompt a call for actions to improve the hearing status and cognitive function of older people to minimize health risks and improve longevity.

## Data Availability Statement

The original contributions presented in the study are included in the article/Supplementary Material, further inquiries can be directed to the corresponding authors.

## Ethics Statement

The studies involving human participants were reviewed and approved by the Research Ethics Committees of Peking University (IRB00001052-13074). The patients/participants provided their written informed consent to participate in this study.

## Author Contributions

S-LZ and W-JK designed the research and directed its implication. JW, DL, and ET prepared and analyzed the data and drafted the manuscript. Z-QG and J-YC contributed to the data management. All co-authors contributed to the manuscript’s modifications and approved the final version.

## Conflict of Interest

The authors declare that the research was conducted in the absence of any commercial or financial relationships that could be construed as a potential conflict of interest.

## Publisher’s Note

All claims expressed in this article are solely those of the authors and do not necessarily represent those of their affiliated organizations, or those of the publisher, the editors and the reviewers. Any product that may be evaluated in this article, or claim that may be made by its manufacturer, is not guaranteed or endorsed by the publisher.

## Abbreviations

HI: hearing impairment
CLHLS: Chinese Longitudinal Healthy Longevity Survey
MMSE: Mini-Mental State Examination
CI: confidence interval
HR: hazard ratio
ADL: activities of daily living.
