# Supplementary material for: Hearing Impairment With Cognitive Decline Increases All-Cause Mortality Risk in Chinese Adults Aged 65 Years or Older: A Population-Based Longitudinal Study
Source: Front Aging Neurosci. 2022 Jun 24;14:865821. doi: 10.3389/fnagi.2022.865821 (PMC9263259; doi:10.3389/fnagi.2022.865821)
Supplement: Supplementary file 1 [file Data_Sheet_1.docx]

**Supplementary figure legends**

**
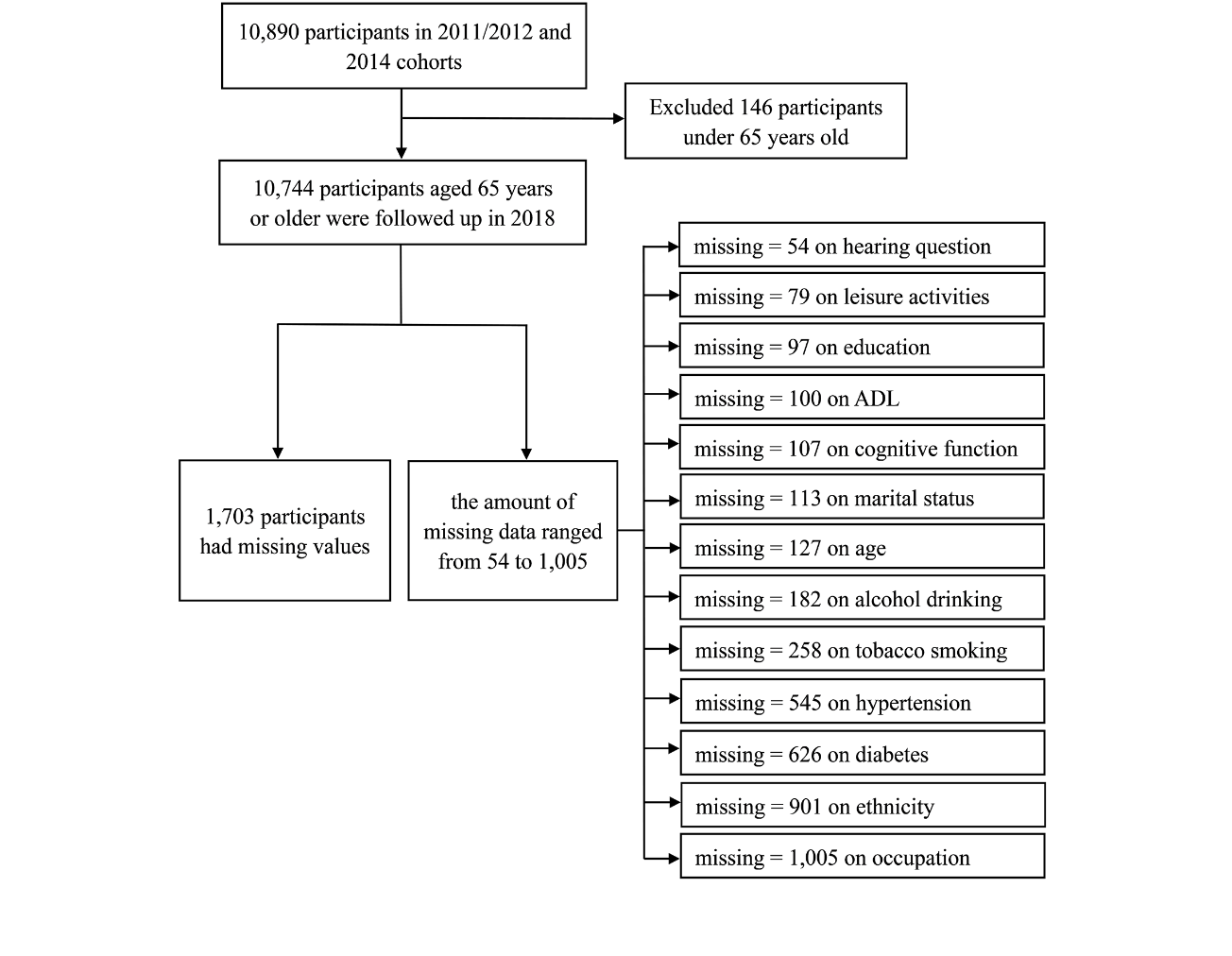
**

**Fig. A1** The characteristics of the raw dataset.

**
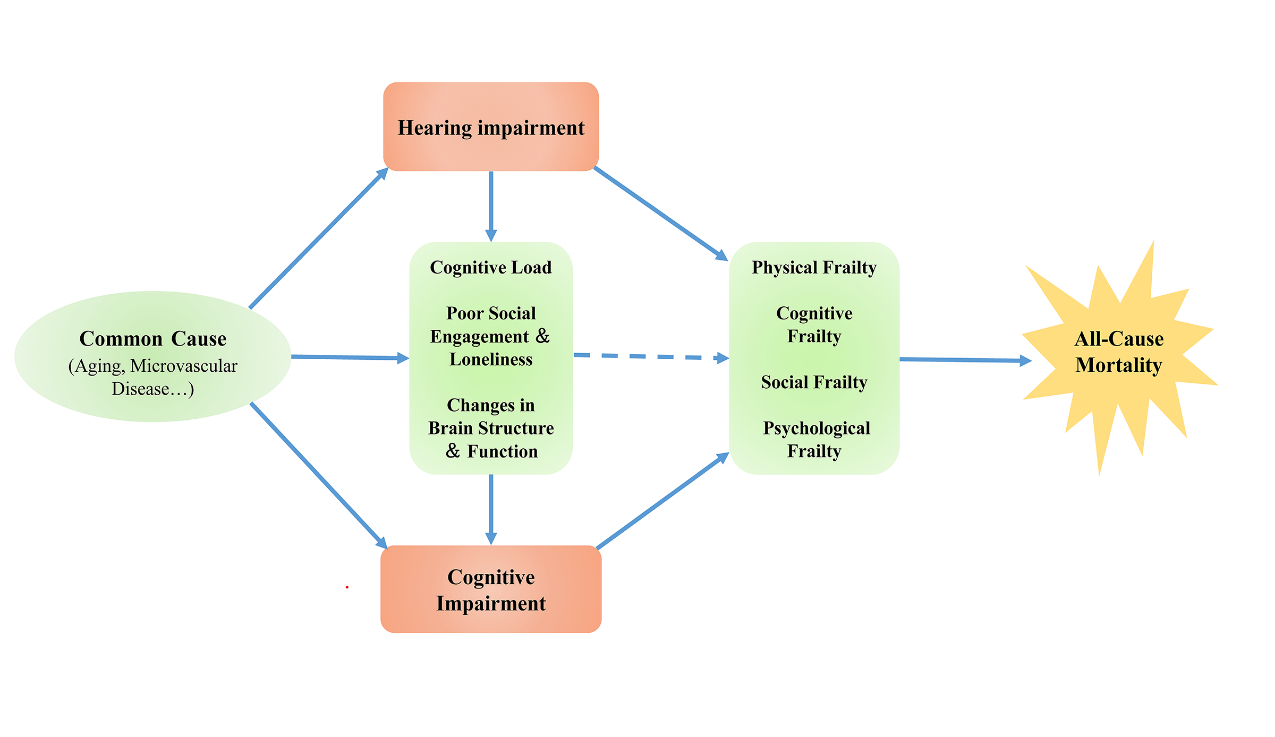
**

**Fig. A2** Proposed hypotheses on the causal direction of the relationship between hearing impairment and cognitive impairment and all-cause mortality.

The left blue arrow includes potential causal paths between HI and cognitive impairment, including:1) increased cognitive load (i.e., information degradation hypothesis), and other effects such as poor social engagement and loneliness; 2) changes to brain structure and function (i.e., sensory deprivation hypothesis); 3) a common cause such as aging or microvascular disease may lead to both HI and cognitive impairment. Further inclusion of HI and cognitive impairment as a result of direct and indirect effects from this causal pathway and may serve as a marker for frailty (i.e., physical, cognitive, social, and psychological frailty, which is a powerful predictor of mortality. It is likely more than one of the pathways depicted may explain the link between HI and cognitive impairment and all-cause mortality. Abbreviation: HI: hearing impairment.
