# Supplementary material for: Hearing Impairment With Cognitive Decline Increases All-Cause Mortality Risk in Chinese Adults Aged 65 Years or Older: A Population-Based Longitudinal Study
Source: Front Aging Neurosci. 2022 Jun 24;14:865821. doi: 10.3389/fnagi.2022.865821 (PMC9263259; doi:10.3389/fnagi.2022.865821)
Supplement: Supplementary file 3 [file Table_2.docx]

**Table A2**. Sensitive analysis of multivariable-adjusted hazard ratios and 95% confidence intervals of all-cause mortality by hearing status (exclude participants who died within half of the year of the follow-up) (*n* = 10196).

| Model | Model 1 | Model 2 | Model 3 | Model 4 | Model 5 |
| --- | --- | --- | --- | --- | --- |
|  | Hazard ratio (95% CI) | | | | |
| **Hearing impairment** |  |  |  |  |  |
| No | - | - | - | - | - |
| Yes | 2.27 (2.13, 2.40) | 1.27 (1.19, 1.35) | 1.15 (1.07, 1.22) | 1.14 (1.07, 1.22) | 1.08 (1.01, 1.15) |
| **Gender** |  |  |  |  |  |
| Female | - | - | - | - | - |
| Male |  | 1.41 (1.32, 1.51) | 1.43 (1.33, 1.55) | 1.44 (1.33, 1.55) | 1.46 (1.35, 1.58) |
| **Age** | - | 1.07 (1.06, 1.07) | 1.05 (1.05, 1.06) | 1.06 (1.05, 1.06) | 1.05 (1.05, 1.06) |
| **Education attainment** |  |  |  |  |  |
| Primary school or higher | - | - | - | - | - |
| None |  | 1.13 (1.05, 1.22) | 1.08 (1.00, 1.16) | 1.08 (1.00, 1.16) | 1.04 (0.96, 1.12) |
| **Residence** |  |  |  |  |  |
| Rural | - | - | - | - | - |
| Urban |  | 1.05 (0.99, 1.12) | 1.07 (1.01, 1.14) | 1.08 (1.01, 1.14) | 1.05 (0.99, 1.12) |
| **Marital status** |  |  |  |  |  |
| Currently married and living with spouse | - | - | - | - | - |
| Others ^a^ |  | 1.26 (1.16, 1.36) | 1.20 (1.11, 1.30) | 1.20 (1.11, 1.30) | 1.18 (1.09, 1.28) |
| **Smoke status** |  |  |  |  |  |
| Never | - | - | - | - | - |
| Ever |  |  | 1.09 (1.01,1.18) | 1.09 (1.01,1.18) | 1.11 (1.03, 1.20) |
| **Drink status** |  |  |  |  |  |
| Never | - | - | - | - | - |
| Ever |  |  | 0.98 (0.91, 1.05) | 0.98 (0.91, 1.05) | 0.98 (1.41, 1.62) |
| **Regular leisure activities** |  |  |  |  |  |
| Yes | - | - | - | - | - |
| No |  |  | 1.73 (1.61, 1.86) | 1.73 (1.60, 1.86) | 1.61 (1.50, 1.74) |
| **ADL** |  |  |  |  |  |
| Don’t need help | - | - | - | - | - |
| Need help |  |  | 1.62 (1.51, 1.73) | 1.61 (1.51, 1.73) | 1.51 (1.41, 1.62) |
| **Self-reported hypertension** |  |  |  |  |  |
| Without | - | - | - | - | - |
| With |  |  |  | 0.98 (0.92, 1.05) | 0.98 (0.92, 1.05) |
| **Self-reported diabetes** |  |  |  |  |  |
| Without | - | - | - | - | - |
| With |  |  |  | 1.32 (1.14, 1.53) | 1.32 (1.14, 1.52) |
| **Cognitive function** |  |  |  |  |  |
| Not impaired | - | - | - | - | - |
| Impaired |  |  |  |  | 1.45 (1.35, 1.55) |

Abbreviations: *CI*: confidence interval; *ADL*: activities of daily living.

Model 1: No variables adjusted.

Model 2: Additionally adjusted for gender, age, education attainment, residence, and marital status based on model 1.

Model 3: Additionally adjusted for smoking status, drinking status, regular leisure activities, and ADL based on model 2.

Model 4: Additionally adjusted for two kinds of diseases (hypertension and diabetes) based on model 3.

Model 5: Additionally adjusted for cognitive function based on model 4.

^a^ ‘Others’ include widowed, separated, divorced and never married.
